# Dissecting the fungal biology of *Bipolaris papendorfii*: from phylogenetic to comparative genomic analysis

**DOI:** 10.1093/dnares/dsv007

**Published:** 2015-04-27

**Authors:** Chee Sian Kuan, Su Mei Yew, Yue Fen Toh, Chai Ling Chan, Yun Fong Ngeow, Kok Wei Lee, Shiang Ling Na, Wai-Yan Yee, Chee-Choong Hoh, Kee Peng Ng

**Affiliations:** 1 Department of Medical Microbiology, Faculty of Medicine, University of Malaya, Kuala Lumpur 50603, Malaysia; 2 Codon Genomics SB, Jalan Bandar Lapan Belas, Selangor Darul Ehsan 47160, Malaysia

**Keywords:** dematiaceous, *Bipolaris papendorfii*, *Cochliobolus*, *Curvularia*, LysM domain

## Abstract

*Bipolaris papendorfii* has been reported as a fungal plant pathogen that rarely causes opportunistic infection in humans. Secondary metabolites isolated from this fungus possess medicinal and anticancer properties. However, its genetic fundamental and basic biology are largely unknown. In this study, we report the first draft genome sequence of *B. papendorfii* UM 226 isolated from the skin scraping of a patient. The assembled 33.4 Mb genome encodes 11,015 putative coding DNA sequences, of which, 2.49% are predicted transposable elements. Multilocus phylogenetic and phylogenomic analyses showed *B. papendorfii* UM 226 clustering with *Curvularia* species, apart from other plant pathogenic *Bipolaris* species. Its genomic features suggest that it is a heterothallic fungus with a putative unique gene encoding the LysM-containing protein which might be involved in fungal virulence on host plants, as well as a wide array of enzymes involved in carbohydrate metabolism, degradation of polysaccharides and lignin in the plant cell wall, secondary metabolite biosynthesis (including dimethylallyl tryptophan synthase, non-ribosomal peptide synthetase, polyketide synthase), the terpenoid pathway and the caffeine metabolism. This first genomic characterization of *B. papendorfii* provides the basis for further studies on its biology, pathogenicity and medicinal potential.

## 1. Introduction

*Bipolaris* is a large genus of dematiaceous hyphomycetes belonging to the order Pleosporales in the class Dothideomycetes.^1^ It is closely related to two other genera, *Cochliobolus* and *Curvularia*. The taxonomy of these three genera has caused confusion for many mycologists as their sexual and asexual states have undergone several nomenclatural changes.^2^ Manamgoda *et al.*^3^ showed that *Cochliobolus* species can be separated broadly into two subgroups, one with *Bipolaris* species and another with *Curvularia* species. Although *Bipolaris* and *Curvularia* share many morphological similarities including the same teleomorph of *Cochliobolus,* some species demonstrate intermediate morphology and some *Bipolaris* species cluster phylogenetically with *Curvularia* species instead of with other *Bipolaris* species.^3^

*Bipolaris* species have been frequently isolated from soil, plant debri, as well as living plants on which they may exist as saprophytes or phytopathogens. The fungal genus *Bipolaris* has a asexual state named *Cochliobolus* are notorious for causing devastating disease epidemics of crop plants such as maize, oat, wheat, rice and sorghum,^4–6^ leading to problems of starvation, malnutrition and world economic crisis.^4,5^ Some plant pathogens such as *Bipolaris australiensis*, *Bipolaris hawaiiensis* and *Bipolaris spicifera* occasionally cause disease in both immunocompetent and immunocompromised humans,^7^ manifesting as allergic or chronic, superficial or deep-seated, localized or disseminated infections.^8^

*Bipolaris papendorfii* has received little attention, compared with other virulent species that are pathogenic in food crops and humans. It is a rare opportunistic pathogen that has been reported in only three cases of keratitis.^8^ Like other plant pathogenic *Bipolaris* species, it causes corn leaf spot,^9^ but the underlying pathogenic mechanism is totally unclear. More interestingly, a recent report described two bioactive compounds, hamigerone and radicinol, from *B. papendorfii* that exhibited anti-proliferative activity in various cancer cells.^10^ Both of these compounds also activated the apoptosis pathway by regulating the expression of BCL-2, caspase 3 and p53.^10^

The development of high-throughput next-generation sequencing technologies (NGS) has provided massive genomic nucleotide sequence data for the study of microbes. Although there are whole genome sequence data for several *Cochliobolus* species in international databases,^6^ the whole genome sequence of *B. papendorfii* has not yet been determined. The genetic fundamental and basic biology of this fungus are largely unknown. In this study, we report the *de nov*o draft genome sequence of *B. papendorfii* UM 226 that was isolated from the skin scraping of a patient with dermatomycosis. The draft genome was generated by a hybrid assembly of Illumina and PacBio sequencing technologies to improve genome assembly. To our knowledge, this report is the first detailed description of the *B. papendorfii* genome. It should provide useful information on the biology, pathogenicity and medicinal potential of this fungus.

## 2. Materials and methods

### 2.1. Ethics statement

The isolate used in this study was obtained from an archived fungal collection. All labels on the source sample have been erased with the exception of sample type and clinical diagnosis. Thus, there is no information traceable back to the patient from whom the isolate was obtained. As such, this genomic study is exempt from ethical approval in our teaching hospital. (http://www.ummc.edu.my/view/content.php?ID=VGxSWlBRPT0=).

### 2.2. Fungal isolate

UM 226 was isolated from the skin scraping of a patient with suspected dermatomycosis in the University of Malaya Medical Centre (UMMC), Kuala Lumpur, Malaysia. The isolate was processed according to the laboratory's standard operating procedures (SOP)^11^ with direct wet mount microscopy followed by inoculation on Sabouraud Dextrose Agar (SDA) for incubation at 30°C up to 7 days, with alternate day examination for fungal growth. Macroscopic examination was carried out to observe colonial characteristics, including color, texture and topography. Tease mount and slide culture were performed to identify the arrangement of conidia and conidiophores. The isolate was viewed under the scanning electron microscope (SEM) to examine wall ornamentation and fine surface features of fungal conidia.

### 2.3. DNA sequencing and phylogenetic analysis

The internal transcribed spacer region (ITS), small subunit of the ribosomal RNA gene (SSU) and large subunit of the ribosomal RNA gene (LSU) were used as targets for molecular identification of the fungal isolate. Total DNA extraction, PCR amplification and sequencing were performed as described previously.^11^ The primers used are listed in Supplementary Table S1. Annealing temperatures used to amplify ITS, SSU and LSU genes were 58, 52 and 46°C, respectively. The sequenced data were subjected to BLASTn search against the non-redundant (nr) NCBI-nucleotide database for fungal identification. Unique ITS, SSU and LSU sequences from the isolate, together with an additional 145 species of *Cochliobolus*, *Bipolaris* and *Curvularia* and an outgroup strain of *Alternaria brassicicola* (Supplementary Table S2) were subjected to phylogenetic analysis. Multiple sequence alignments of collected ITS, LSU and SSU sequences were generated using M-Coffee.^12^ Individual alignments were concatenated for Bayesian Markov Chain Monte Carlo (MCMC) analysis partitioned by gene. Bayesian tree analyses were performed using MrBayes v3.2.1^13^ with reversible jump MCMC averaging over the entire general time reversible (GTR) rates and gamma-distributed rate heterogeneity for all subsets of partitioned scheme. A total of 5,000,000 generations were run with a sampling frequency of 100, and diagnostics were calculated for every 1,000 generations. The first 12,500 trees were discarded with burn-in setting of 25%. Convergence was assessed with a standard deviation of split frequencies below 0.01, no obvious trend for the plot of the generation versus the log probability of the data, and the potential scale reduction factor (PSRF) close to 1.0 for all parameters.

### 2.4. Genomic DNA extraction

Genomic DNA was extracted from fresh fungal mycelia in accordance with the protocol of Moslem *et al.*^14^ with slight modification. Briefly, mycelia were scraped off from the agar surface and crushed into fine powder with liquid nitrogen. One gram of crushed fungal powder was suspended in 6 ml of DNA extraction buffer [200 mM Tris–HCl, (pH 8.5), 250 mM NaCl, 25 mM EDTA, 0.6% (w/v) SDS] in a 50-ml centrifuge tube and incubated at 65°C for 10 min. Next, 1.4 ml of 3 M sodium acetate (pH 5.3) was added to the mixture and followed by incubation at 80°C for 20 min. The tube was then centrifuged at 4,000 × *g* for 5 min and the supernatant was transferred into a new 50-ml centrifuge tube. Genomic DNA was then precipitated with equal volume of 100% (v/v) isopropanol. Genomic DNA was washed from the pellet by adding 500 µl of ice-cold 70% (v/v) ethanol and finally dissolved in nuclease-free distilled water. The purified genomic DNA was treated with RNAase at 37°C for overnight. Then, the mixture was incubated at 65°C for 10 min, followed by centrifugation at 28,433 × *g* for 5 min. The supernatant was transferred into a new 1.5-ml microcentrifuge tube. The quantity and quality of the extracted genomic DNA were determined using NanoDrop 2000c spectrophotometer (Thermo Fisher Scientific).

### 2.5. Genome sequencing and *de novo* hybrid assembly

Illumina library was prepared using TruSeq v3 Reagent Kits (Illumina). UM 226 genomic DNA was sheared into smaller fragments by Covaris S/E210 or Bioruptor. The 500-bp fragments were purified though gel electrophoresis, which then selectively enriched and amplified by PCR. Next, qualified sequencing library were sequenced using Illumina HiSeq 2000 Sequencer (Illumina) with 2 × 90 bp paired-end mode.

PacBio RS sequencing was performed using the PacBio RS II system (Pacific Biosciences). UM 226 genomic DNA was used for 10-kb library preparation using PacBio DNA Template Prep Kit 2.0 (3–10 kb). SMRTbell templates were then annealed using PacBio DNA/Polymerase Binding Kit P4. Three SMRT cells were used for sequencing with MagBead standard Seq v2 collection protocol using 180-min movies.

Genome assembly was performed using the hybrid approach using ECtools^15^ with Illumina contigs and PacBio long reads. Illumina short reads were first pre-processed trimming bases with a Phred quality below Qv20 from the 3′ end of the reads, retaining reads ≥ 50 bp and reads with 40% bases having Qv ≤20 were filtered out using FASTX-Toolkit (http://hannonlab.cshl.edu/fastx_toolkit/). The pre-processed reads were corrected using Quake software^16^ with 16-mer setting. Corrected reads were then assembled into preliminary contigs using Velvet version 1.2.0715 with k-mer setting = 57, scaffolding = no, insert_length = 462 and ins_length_sd = 60.

ECTools then utilize velvet assembled contigs with length ≥500 bp to correct errors of the PacBio long reads. Error corrected long reads were then assembled using Celera assembler.^17^ Both ECTools and Celebra assembly were performed according to the software instruction with default parameters setting. Celera assembled contigs smaller than 10 kb were discarded, and Quiver was used to polish the remaining contigs. The assembly was imported into SMRT analysis software version 2.1.1 through SMRT portal interface as the reference, and three SMRT cells data generated were aligned to the genome using the RS_Resequencing protocol iteratively. Through the polishing steps, low-quality contigs with low coverage information from Quiver output were discarded from the final assembly.

### 2.6. Gene prediction and annotation

Protein coding sequences of *B. papendorfii* UM 226 were predicted using GeneMark-ES version 2.3e.^18^ Annotation of coding sequences for UM 226 was completed using BLAST (Basic Local Alignment Search Tool) searches against the NCBI nr protein and SwissProt databases. Gene Ontology and KEGG metabolic pathways matches were carried out using local BLAST2GO tools.^19^ Protein classifications were performed by EuKaryotic Orthologous Group (KOG).^20^ The total number of predicted proteins involved in post-translational modification, protein turnover, chaperones (KOG annotation for other *Cochliobolus* species) was obtained from the Joint Genome Institute (JGI) website, http://genome.jgi.doe.gov/. Protein domain families were matched to Pfam database using InterProScan 5.^21^ Individual rRNA and tRNA sequences were identified using RNAmmer v1.2^22^ and tRNAscan-SE v1.3.1,^23^ respectively. Putative transposable elements were identified using Transposon-PSI (http://transposonpsi.sourceforge.net).

Carbohydrate-Active enzymes (CAZymes) were functionally annotated by submitting the predicted protein models to the databases of automated Carbohydrate-active enzyme ANnotation (dbCAN).^24^ Small secreted proteins were identified by the prediction of cleavage sites and signal peptide/non-signal peptide using SignalP version 4.1^25^ after discarding proteins with transmembrane domains that were determined by TMHMM version 2.0.^26^ However, one transmembrane domain located at in the N-terminal 40 amino acids is allowed, because the domain is responsible to the secretion signal. Genomic mapping of fungal secondary metabolite clusters was performed using web-based SMURF (Secondary Metabolite Unknown Regions Finder) (www.jcvi.org/smurf/).^27^ Amino acid sequences with e-value threshold ≤1e−5, alignment length over 70% of its own length and over 50% match identity were selected and assigned as the annotation of predicted genes.

### 2.7. Orthologous genes and comparative genomic analysis

The protein sequences of all current publicly available *Cochliobolus* genomes (*Cochliobolus heterostrophus* C5,^6,28,29^ *C. heterostrophus* C4,^4,30,31^ *C. carbonum* 26-R-13,^6,29^ *C. victoriae* FI3,^6,29^ *C. miyabeanus* ATCC 44560,^6,29^ *C. sativus* ND90Pr^4,30,31^ and *C. lunatus* m118^29^) were used to determine the orthologous genes in *B. papendorfii* UM 226. The genome sequences of the seven *Cochliobolus* species were downloaded from JGI. The OrthoMCL version 2.02^32^ was used in the analysis of protein sequences clustering (≥33 amino acids) for *B. papendorfii* UM 226 and the seven genome references by all-against-all BLASTp searches of all proteins. The reciprocal best hits from distinct genomes were identified as orthologous genes.

### 2.8. Phylogenomic analysis

A phylogenomic tree was built using all proteome clusters of the seven *Cochliobolus* species generated from comparative analysis. The tree is rooted with *A. brassicicola* ATCC 96836 as outgroup. A total of 6,051 single-copy orthologous genes containing one member in each species was subjected to individual sequence alignments using ClustalW version 2.0.^33^ All spurious sequences or poorly aligned regions were discarded with trimAL (with –gt 0.5). The filtered multiple alignments were then concatenated into a superalignment with 2,991,675 characters. Bayesian phylogenetic analysis was run using MrBayes v3.2.1 with mixed amino acid model, gamma-distributed rate variation across sites and a proportion of invariable sites. The MCMC was run using a sampling frequency of 100 for 100,000 generations with a burn-in setting of 25%.

### 2.9. Prediction of secondary structure and homology modelling

PSIPRED v3.3^30^ was used to predict the secondary structure of the LysM domains. Three-dimensional structural models of the LysM domains were generated using SWISS-MODEL server.^31^ Amino acid sequence alignment between UM226_683 domains and Ecp6 was performed using ClustalW version 2.0. The template used in this modelling was selected from template identification function in the SWISS-MODEL program. In this study, a *Cladosporium fulvum* LysM effector Ecp6 (Protein Data Bank entry 4b8v)^34^ was selected as the template. The predicted models were stored as a PDB output file, and the structures were visualized by Swiss-PdbViewer 4.0.1.^35^ The quality of the protein model generated was validated by using the PROCHECK program as described previously.^36^

### 2.10. Mating type locus identification and phylogenetic analysis

*MAT1-2* mating type regions immediately 5 kb upstream and downstream were retrieved from the sequenced genome using Artemis v12.0 sequence viewer.^37^ Bayesian tree analysis was carried out by using a procedure similar to ribosomal molecular identification with 500,000 generations run. A phylogenetic tree was constructed using *MAT1-2* nucleotide sequences from *B. papendorfii* UM 226, together with additional nine species of *Cochliobolus* and *Bipolaris*. The tree is rooted with *Alternaria alternata* as outgroup.

## 3. Results and discussion

### 3.1. Morphological and molecular identification

**Figure 1.**
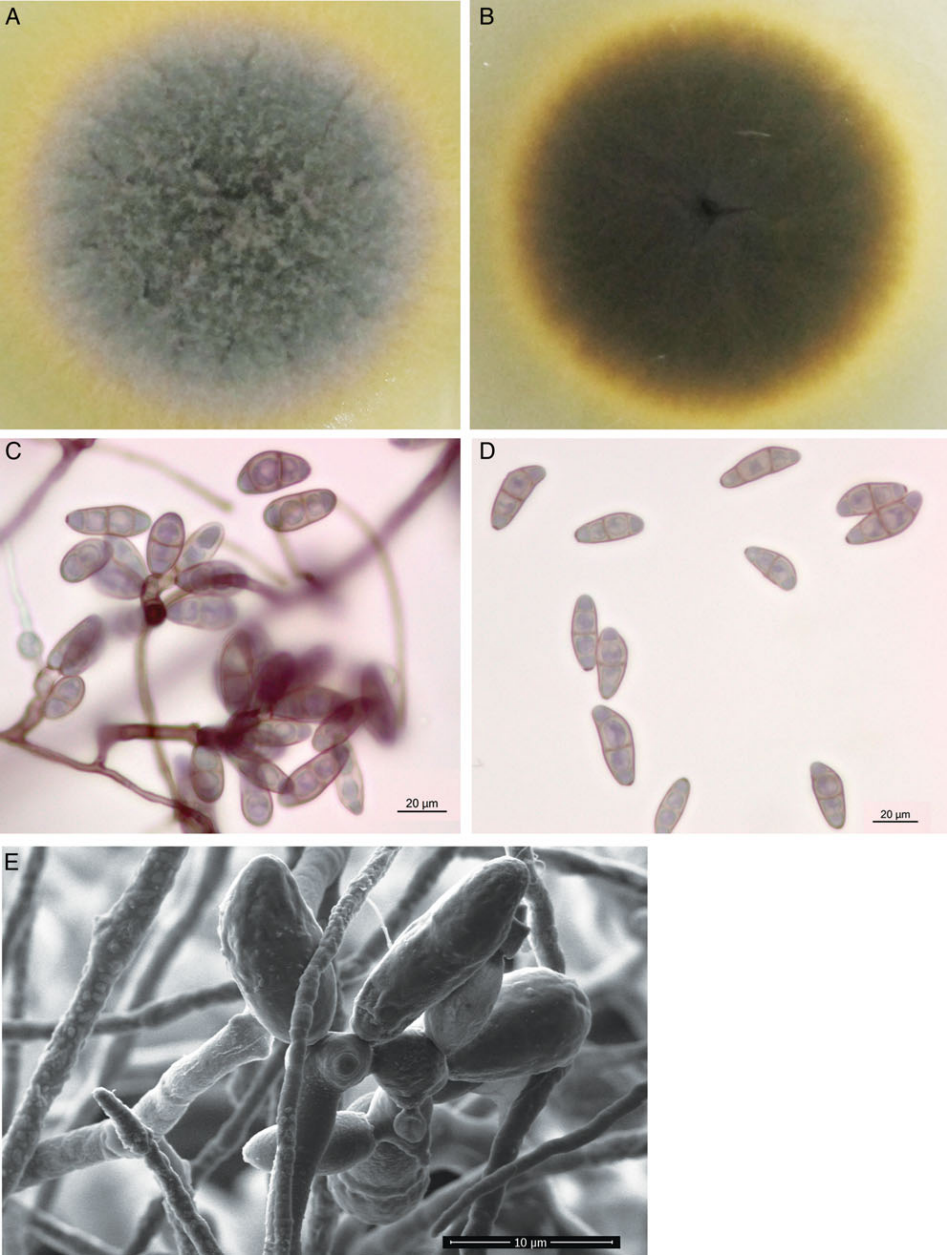
Colonial characteristic and microscopic morphology of *Bipolaris papendorfii* UM 226. The surface (A) and reverse (B) colony morphology of *B. papendorfii* UM 226 after being cultured for 7 days. Light micrograph showing (C) typical zig-zag conidiophore with several conidia and (D) conidia with three pseudoseptates (×400 magnification, bars 20 µm). Scanning electron micrograph showing (E) zig-zag conidiophores with verruculose walled conidia (×3,065 magnification, bars 10 µm). This figure is available in black and white in print and in colour at *DNA Research* online.

The *B. papendorfii* UM 226 colonies on SDA were fast growing, velvety and compact with abundant short aerial mycelium (Fig. 1a). The surface of the colonies was initially grayish white to olivaceous green and becomes black with a raised grayish periphery after being cultured for 7 days (Fig. 1a). It had a black-green coloration on the reverse side (Fig. 1b). The diameter of the colony was 60 mm after 7 days of incubation at 30°C. Microscopic morphology demonstrated simple or flexuose branched, pale to medium brown conidiophores (up to 200 µm long, 4–9 µm wide) with zig-zag rachis bearing conidium in a sympodial pattern (Fig. 1c). Conidiogenous nodes were verruculose, distinct, swollen and up to 5 µm wide (Fig. 1d). The conidia (24–40 × 10–20 µm) were curved, kidney shaped or obpyriform (Fig. 1c). The conidia had three pseudoseptates with the widest at the second cell from the base. SEM observation demonstrated typical, roughed, zig-zag conidiophores with several conidia (Fig. 1e). Unlike other *Bipolaris* spp. that have typical smooth walled conidia,^8^ the SEM microphotograph of the UM 226 conidia showed that they were verrucose walled with thickenings at places of pseudosepta (Fig. 1e). On the basis of these characteristics, the UM 226 clinical isolate was found to be morphologically related to *B. papendorfii*.^8^

**Figure 2.**
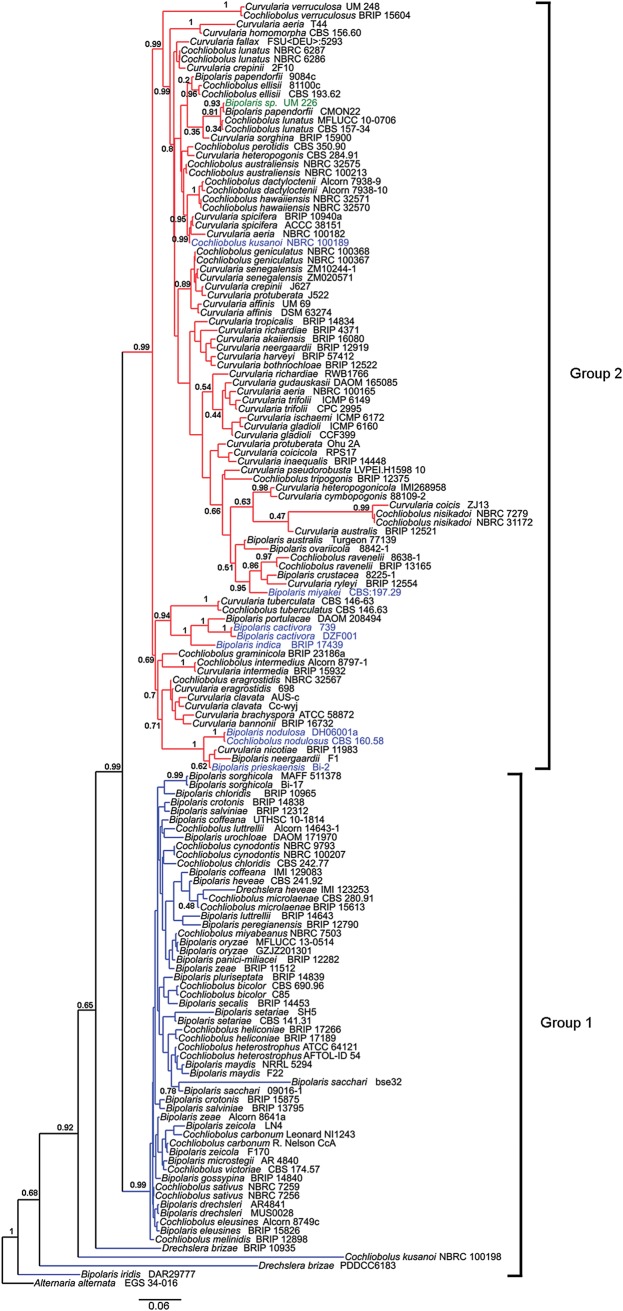
Bayesian phylogram generated based on combined genes of ITS, SSU and LSU sequenced data. The tree is rooted with *Alternaria brassicicola* as outgroup. Numbers on the nodes indicate Bayesian posterior probability based on 100 sampling frequency for a total of 150,000 generations. This figure is available in black and white in print and in colour at *DNA Research* online.

The preliminary morphological identification of the UM 226 isolate was confirmed by PCR amplification of the ITS, SSU and LSU gene regions, which yielded specific amplicons of ∼540 (ITS), 1,200 (SSU) and 900 bp (LSU), respectively (Supplementary Fig. S1). The sequenced data were used to construct a phylogram using combined gene analysis with the additional 145 ex-type strains of *Cochliobolus*, *Bipolaris* and *Curvularia* spp. (Fig. 2). As previously noted,^2^ the phylogenetic tree analysis separated the *Curvularia*, *Bipolaris* and *Cochliobolus* species into two major, well-supported groups: Groups 1 and 2 (Fig. 2). Phylogenetic analysis of the combined dataset of the three loci revealed that 59 species were clustered in Group 1 and 87 species were clustered in Group 2. Group 1 consisted of *Bipolaris* and *Cochliobolus* spp. while Group 2 comprised species of *Curvularia*, *Bipolaris* and *Cochliobolus*. All the plant pathogenic and economically important fungi are identified in genus *Bipolaris*, which were grouped into Group 1.^2^ The *Bipolaris* species that clustered in Group 2 (Fig. 2). Multilocus phylogenetic analysis revealed that UM 226 was clustered together with *B. papendorfii* strain CMON22 in Group 2 (Fig. 2). Based on the morphological examination and multigene phylogeny, the isolate was identified as *B. papendorfii*.

### 3.2. Genome features of *B. papendorfii* UM 226

In this study, Illumina HiSeq 2000 and PacBio sequencing technologies were used to produce hybrid genome assembly for *B. papendorfii* UM 226. The total assembly size of *B. papendorfii* UM 226 genome is 33.4 Mb. The sequencing reads were assembled into 374 contigs with an N50 contig size of 146,099 kb. Hybrid assembly of *B. papendorfii* UM 226 showed a lower number of contigs and a higher N50 value (Table 1). Summary of the sequence statistics is presented in Table 1. The genome had an average G + C content of 50.65%.

**Table 1.** Genome statistics of *Bipolaris papendorfii* UM 226

Details	Illumina HiSeq 2000	PacBio RS II system	Hybrid assembly of Illumina and PacBio
Sequencing depth	∼87×	∼34×	—
Read type	Small insert	Single end	—
Library size (bp)	500	20,000	—
Read length (bp)	90	2,742	—
Total number of reads	33,655,556	405,257	—
Total number of bases	3,029,000,040	1,111,215,948	—
Assembly size (Mb)	33,620,574	—	33,379,470
Contigs (≥500 bp)	730	—	374
Contigs (N50) (kb)	127,284	—	146,099
Contigs G + C (%)	50.55	—	50.65

### 3.3. Transposable elements

Transposable elements (TEs), also known as jumping genes, were important in the variability, gene structure and evolution of the genome. Recently, Santana *et al.*^38^ demonstrated that *C. heterostrophus* race O genome (∼36 Mb) harbours 5.9% of TEs. Here, our bioinformatics prediction revealed that 2.49% (831,552 bp) of the assembled genome of *B. papendorfii* UM 226 consisted of TEs (Table 2). The transposable elements of Class I, retrotransposons comprised 2.31% of the genome, whereas Class II transposable elements, DNA transposons comprised only 0.19% of the genome. LINE (Long Interspersed Nuclear Element)- and LTR (Long Terminal Repeat)-type retrotransposons are mostly presented in the fungal genomes.^39,40^ Gypsy and Copia elements are the LTR superfamilies that widely distributed in these genomes. As previously noted,^38^ Gypsy elements were identified to be located upstream of the *PKS1*, *OXI1* and *LAM1* genes, downstream of *PKS2* gene as well as between the *RED2* and *RED3* genes in the *C. heterostrophus* race O genome; Copia element was located downstream of the *TOX9* gene in the genome. In this study, our analysis showed that 0.24, 0.67 and 0.29% of the sequenced *B. papendorfii* UM 226 genome, estimated 33.4 Mb, consists of LINE, gypsy and TY1_Copia, respectively. However, the locations of these TEs in the genome remain unknown at this stage of knowledge. The most predominant TE families, both in terms of number of copies and element size, are DDE_1 transposases (Table 2). The DDE_1 transposases contain a functional domain to mediate DNA-binding, protein–protein interaction and ‘cut-and-paste’ activities.^41^ The DDE domain has been identified in all eukaryotic cut-and-paste TEs, suggesting their common evolutionary origin.^42^

**Table 2.** Putative transposable elements in the genome sequence of *Bipolaris papendorfii* UM 226

Class	Family name	Total number	Total bases	Percentage of assembled genome
I	DDE_1	422	369,615	1.11
	gypsy	279	223,716	0.67
	LINE	54	78,738	0.24
	ltr_Roo	2	348	0.00001
	TY1_Copia	87	95,805	0.29
II	helitronORF	4	2,673	0.01
	hAT	21	15,879	0.05
	mariner	55	36,201	0.11
	mariner_ant1	6	2,793	0.01
	MuDR_A_B	19	4,701	0.01
	cacta	10	1,083	0.00003
	Total		831,552	2.49

### 3.4. Gene prediction

Our pipeline showed that there are 128 tRNAs, 12 5S rRNA, 3 18S rRNA and 3 28S rRNA in the genomic sequence. In addition, the *B. papendorfii* UM 226 genome revealed a total of 11,015 putative coding DNA sequences (CDS) with an average gene length of 1,425 bp. Gene density of protein coding genes is 3.29 genes/10 kb. The genome size of *B. papendorfii* UM 226, number of predicted genes and gene density are comparable to the previously sequenced *Cochliobolus* species genomes (Supplementary Table S3). On average, there are 2.95 exons per gene. A total of 10,708, 6,963 and 8,283 gene-coding sequences are homologous to currently available proteins in the NCBI nr, SwissProt and InterPro databases with 7,759 hypothetical proteins identified based on the top hit of the BLAST result against NCBI nr database. The large amount of hypothetical proteins in the genome indicated the uniqueness of *B. papendorfii* isolate.

**Figure 3.**
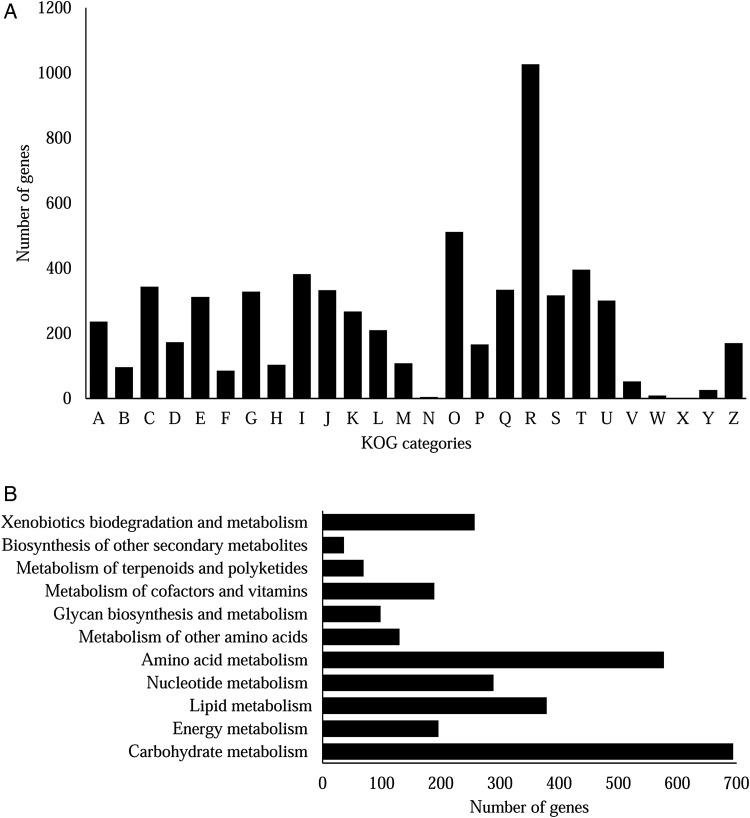
KOG and KEGG classifications of proteins in *Bipolaris papendorfii* UM 226 (A) KOG class annotation distribution of *B. papendorfii* UM 226 genome. A, RNA processing and modification; B, Chromatin structure and dynamics; C, Energy production and conversion; D, Cell cycle control, cell division, chromosome partitioning; E, Amino acid transport and metabolism; F, Nucleotide transport and metabolism; G, Carbohydrate transport and metabolism; H, Coenzyme transport and metabolism; I, Lipid transport and metabolism; J, Translation, ribosomal structure and biogenesis; K, Transcription; L, Replication, recombination and repair; M, Cell wall/membrane/envelope biogenesis; N, Cell motility; O, Post-translational modification, protein turnover, chaperones; P, Inorganic ion transport and metabolism; Q, Secondary metabolites biosynthesis, transport and catabolism; R, General function prediction only; S, Function unknown; T, Signal transduction mechanisms; U, Intracellular trafficking, secretion, and vesicular transport; V, Defense mechanisms; W, Extracellular structures; X, Unnamed protein; Y, Nuclear structure and Z, Cytoskeleton. (B) Distribution of predicted proteins from *B. papendorfii* UM 226 genome that involved in in metabolic pathway by KEGG database.

To further characterize the predicted proteins, the genome was mapped to KOG and KEGG databases. All the proteins were ascribed to 26 different functional groups based on KOG classification (Fig. 3a and Supplementary Table S4). Apart from the poorly characterized categories: categories [R] General functions prediction only, [S] Function unknown and [X] Unnamed protein, the top five KOG groups they were annotated in were [O] Post-translational modification, protein turnover, chaperones (512 genes), [T] Signal transduction mechanisms (396 genes), [I] Lipid transport and metabolism (382 genes), [C] Energy production and conversion (344 genes) and [Q] Secondary metabolites biosynthesis, transport and catabolism (334 genes) (Fig. 3a). Like other *Cochliobolus* species [*C. heterostrophus* C4 (560 copies), *C. heterostrophus* C5 (608 copies), *C. victoriae* F13 (597 copies), *C. sativus* ND90Pr (585 copies), *C. carbonum* 26-R-13 (597 copies), *C. lunatus* m118 (587 copies)] that have been previously sequenced by JGI, the majority of the genes in *B. papendorfii* UM 226 genome was involved in post-translational modifications (Fig. 3a). Post-translational modifications of proteins regulate a wide variety of basic cellular functions in fungi, such as cellular growth, adaptive and developmental processes.^43^ Autophagy plays a vital role in cellular growth, degradation as well as cell survival in filamentous fungi, especially in starvation condition.^44^ Interestingly, we identified 103 putative serine/threonine-protein kinase involved in autophagy from the KOG group O (Supplementary Table S4). Moreover, various fungal cell surface proteins post-translationally modified in fungi are involved in fungal virulence.^43^ To gain insight into the gene functions, we managed to assign 3,074 predicted proteins to their orthologous genes in the KEGG database. At the first glance, there were 1,125 predicted proteins of *B. papendorfii* UM 226 involved in metabolic pathways in the KEGG database (Supplementary Table S5). The top five categories in KEGG metabolic pathway are carbohydrate metabolism, amino acid metabolism, lipid metabolism, nucleotide metabolism and xenobiotics biodegradation and metabolism (Fig 3b). There are 293 unique reactions corresponding to carbohydrate metabolism. This result suggests that there are enriched carbohydrate mechanisms such as glycolysis, gluconeogenesis and citrate cycle (TCA cycle), that can facilitate the use of diverse substrates of *B. papendorfii* UM 226 in exposure to various carbohydrate sources in the environment.

### 3.5. The CAZymes family

**Figure 4.**
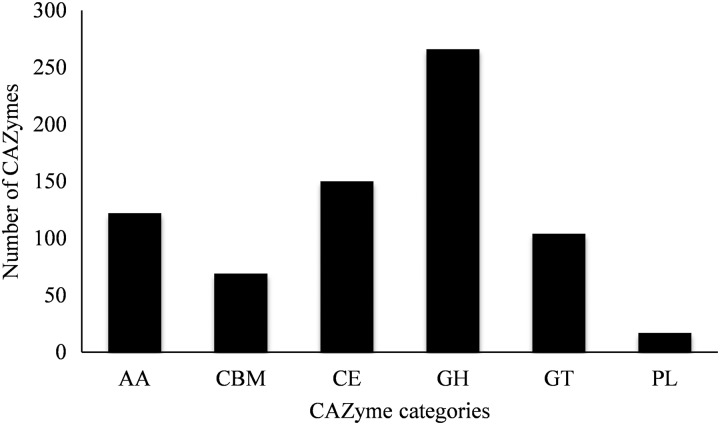
CAZyme class annotation distribution of *Bipolaris papendorfii* UM 226 genome. AA, auxiliary activities; CBM, carbohydrate-binding module; CE, carbohydrate esterase; GH, glycoside hydrolases; GT, glycosyltransferase; PL, polysaccharide lyase.

All types of CAZymes, especially plant cell wall degrading enzymes, are commonly produced by fungal plant pathogens to penetrate the rigid barrier of the plant cell wall.^45^ Most of the fungal CAZymes were known to degrade plant cell wall materials to obtain carbon source for fungal growth as well as for penetration and infection of their host plants.^45^ As *B. papendorfii* has been reported to cause corn infection,^9^ the *B. papendorfii* UM 226 genome was mapped to the CAZy database to determine the presence of CAZyme-coding homologous genes. A total of 728 putative CAZyme-coding homologous genes were determined in the genome. This comprises 122 with auxiliary activities (AA), 69 carbohydrate-binding module (CBM), 150 carbohydrate esterases (CE), 266 glycoside hydrolases (GH), 104 glycosyl transferases (GT) and 17 polysaccharide lyases (PL) (Fig. 4 and Supplementary Table S6). The number of GH, GT and PL family candidates identified are comparable to the several reported in several studies for necrotrophic and hemibiotrophic *Cochliobolus* species.^28,45^ The number of GHs in *C. heterostrophus* C4, *C. heterostrophus* C5 and *C. sativus* ND90Pr are 276, 292 and 272, respectively; the number of GTs in *C. heterostrophus* C4, *C. heterostrophus* C5 and *C. sativus* ND90Pr are 96, 103 and 99, respectively; the number of PLs in *C. heterostrophus* C4, *C. heterostrophus* C5 and *C. sativus* ND90Pr are 15, 15 and 15, respectively.^28,44^ Interestingly, the *B. papendorfii* UM 226 genome contains a higher number of CE-coding gene homologs (150 copies) compared with that of the reported *Cochliobolus* species, such as *C. heterostrophus* C4 (46 copies), *C. heterostrophus* C5 (49 copies) and *C. sativus* ND90Pr (47 copies).^28,45^ Our findings revealed that the fungus has 12 of the 16 CE families, with family CE2, CE6, CE11 and CE13 missing. The high number of CE1 (42 copies) and CE10 (57 copies) indicated that *B. papendorfii* UM 226 might produce many putative plant degrading enzymes containing carboxylesterase and endo-1,4-β-xylanase activities, respectively.^45^ CE1 and CE10 members were reported to have a wide range of substrate specificities. CE1 and CE10 are hemicellulose-targeting CEs.^46,47^ In this study, we identified seven copies of putative endo-1,4-β-xylanases, which are responsible to degrade the 1,4-glycosidic bond and thus depolymerize the xylan polymer (a major carbohydrate component of hemicellulose) into its monomer constituents (Supplementary Table S6).^48^ Our result also demonstrated a high number of GH and AA genes, postulating the ability of our fungal isolate to degrade lignocellulose, which is a main structural constituent of non-woody or woody plants. A total of 16 putative ligninolytic genes: multicopper oxidase (6 copies) and heme peroxidase (10 copies) were identified in this work.^49,50^ Multicopper oxidases and heme peroxidases involved in lignin degradation by oxidizing lignin subunits using molecular oxygen (electron acceptor) and extracellular hydrogen peroxide (co-substrate), respectively.^48,49^ Collectively, *B. papendorfii* UM 226 possesses a wide array of CAZymes that are involved in the degradation of the complex carbohydrates in the plant cell walls.

### 3.6. Comparative genomics of *B. papendorfii* UM 226 and other *Cochliobolus* species

**Figure 5.**
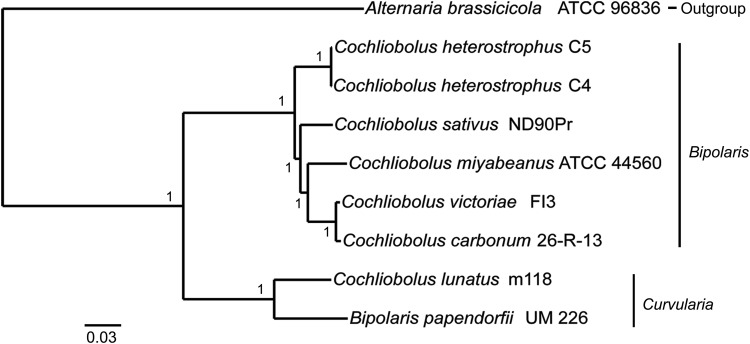
Comparative phylogenomic analysis of *Bipolaris papendorfii* UM 226 along with seven previously published *Cochliobolus* genomes by using Bayesian analysis. Number at the node referring to Bayesian posterior probability. The tree is rooted with *Alternaria brassicicola* ATCC 96836 as outgroup.

In this work, we selected seven publicly available *Cochliobolus* species genomes and an *A. brassicicola* genome for rooting the phylogenomic tree (Fig. 5). A total of 101,438 proteins were clustered into 14,296 orthologous clusters with 6,051 single-copy orthologous genes determined. It should be noted that *B. papendorfii* UM 226 was tightly clustered with *C. lunatus* m118. Among the 14,296 gene family clusters, 222 gene families were conserved between *B. papendorfii* UM 226 and *C. lunatus* m118. In agreement with the multilocus phylogenetic analysis (Fig. 2), these data showed a close evolutionary relationship between *B. papendorfii* UM 226 and *Curvularia* species, which are separated from all plant pathogenic *Cochliobolus* (anamorph *Bipolaris*) species (Fig. 5).

**Figure 6.**
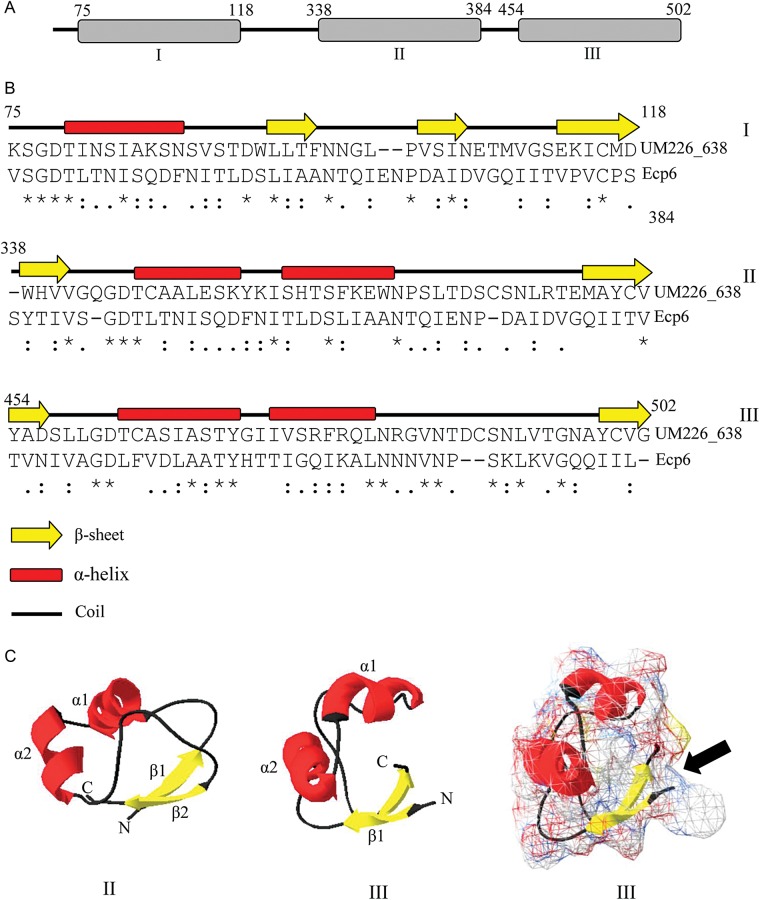
LysM domain of *Bipolaris papendorfii* UM 226_683 hypothetical protein. (A) Schematic representation of the locations of LysM domains in UM226_683 hypothetical protein. I, II and III represent the LysM domain I, II and III. (B) Secondary structure prediction of the LysM domains. Amino acid sequence alignment between UM 226_683 domains and Ecp6 was performed using ClustalW version 2.0. Asterisk (*) indicates positions that have a single, fully conserved residue; colon (:) indicates conservation between groups of strongly similar properties; period (.) indicates conservation between groups of weakly similar properties. (C) Three-dimensional ribbon structures of the LysM domains. Panels 1 and 2 show LysM domain II and III, respectively. Panel 4 displays the molecular surface of LysM domain III. The arrow in panel 4 shows the shallow groove described as binding site of chitin oligomers.^52^ α-helices are shown in red; β-sheets are shown in yellow and random coils are shown in black. The N- and C-terminal ends are labelled.

Whole proteome alignment of the *B. papendorfii* UM 226 and the seven *Cochliobolus* gene contents indicated that *B. papendorfii* UM 226 contained 37 gene family clusters composed of 83 unique genes. All of these unique genes encode for hypothetical proteins or unknown proteins with no apparent homologs to the currently existing gene sequences deposited in NCBI nr and SwissProt databases. Protein domain analysis revealed that seven of the family clusters were related to resistance-associated proteins, retrotransposon, peptidoglycan-binding function, hydrolytic functions, aspartic peptidase and DNA-binding function (Table 3). Among them, particular attention was paid to a hypothetical protein (UM226_638) with multiple lysin motifs (LysMs) domains. Based on the CAZyme annotation, UM226_638 hypothetical protein belongs to the carbohydrate-binding module (CBM50) of CAZyme which is involved in the degradation of chitin or peptidoglycan. Fungal LysM effectors had been shown to have chitin-binding affinity to perturb chitin-induced immunity from their host plant.^51^ A novel virulence factor with LysM domains, known as Ecp6 effector, was well characterized from the tomato pathogenic fungus *C. fulvum*.^52^ The presence of LysM domains in Ecp6 effector was postulated to protect fungal cell walls from plant chitinases hydrolysis by competing with host receptors for chitin.^52^ As previously noted,^52^ the LysMs-containing UM226_638 protein belongs to type A fungal LysM protein (PF01467) and contains three LysM domains. Many of the type A LysM proteins play an important role in infection process.^53^ For convenient description hereafter, these three domains were assigned as LysM domain I (75–118 amino acids), LysM domain II (338–384 amino acids) and LysM domain III (454–502 amino acids) (Fig. 6a). Unlike *C. fulvum* secreted Ecp6,^38,39^ we found that the UM226_638 protein do not have a signal peptide for secretion. Thus, UM226_638 protein might not be a secreted protein. Moreover, secondary structure prediction showed that only LysM domain II and domain III have a βααβ secondary structure (Fig. 6b). On the contrary, LysM domain I composed of three α-helices and a β-sheet (Fig. 6b).

**Table 3.** Specific family clusters in *Bipolaris papendorfii* UM 226

Families	Gene ID	InterPro ID//domain
Bipap11034	UM226_39	IPR023213//Chloramphenicol acetyltransferase-like domain IPR003480//Transferase
	UM226_347	IPR023213//Chloramphenicol acetyltransferase-like domain
	UM226_1504	IPR023213//Chloramphenicol acetyltransferase-like domain IPR003480//Transferase
Bipap12004	UM226_638	IPR018392//LysM domain
	UM226_6391	IPR018392//LysM domain
Bipap11994	UM226_10258	IPR000383//Alpha/Beta hydrolase fold IPR005674//CocE/NonD hydrolase IPR008979//Galactose-binding domain-like IPR013736//Peptidase S15/CocE/NonD, C-terminal IPR029058//X-Pro dipeptidyl-peptidase-like domain
	UM226_11642	IPR000383//Alpha/Beta hydrolase fold IPR005674//CocE/NonD hydrolase IPR029058//X-Pro dipeptidyl-peptidase-like domain
Bipap10443	UM226_9098	—
	UM226_9156	—
	UM226_10550	IPR005162//Retrotransposon gag domain
Bipap12068	UM226_9099	IPR021109//Aspartic peptidase domain
	UM226_10987	IPR021109//Aspartic peptidase domain
Bipap12005	UM226_6690	IPR006600//HTH CenpB-type DNA-binding domain
	UM226_9523	IPR006600//HTH CenpB-type DNA-binding domain IPR004875/DDE superfamily endonuclease, CENP-B-like
Bipap11992	UM226_10253	IPR029068//Glyoxalase/Bleomycin resistance protein/Dihydroxybiphenyl dioxygenase
	UM226_11078	IPR029068//Glyoxalase/Bleomycin resistance protein/Dihydroxybiphenyl dioxygenase

Tertiary structures of the LysM domain II and LysM domain III were then generated by using the SWISS-MODEL server based on the experimentally solved structural homolog. As previously reported,^38,39^ the three-dimensional structural prediction showed that the domains comprised two α-helices packed on the same side of the anti-parallel two β-sheets (Fig. 6c, panel 1 and 2). The molecular surface of the third LysM domain of UM226_638 was computed (Fig. 6c, panel 3). In consistent with the structural homology of *C. fulvum* Ecp6,^52^ a shallow groove was observed in the LysM domain III that provides a binding site for chitin oligomers. Accuracy of the domain models generated was verified by PROCHECK program, a protein structure validation program (data not shown). The stereochemical parameters were all indicated to be within the acceptable limit, and it can serve as a good hypothetical domain structures for further functional study. Taken together, the putative unique LysMs-containing UM226_638 protein might be a fungal avirulence protein that involved in fungal virulence on its host.

### 3.7. Sexual reproduction

**Figure 7.**
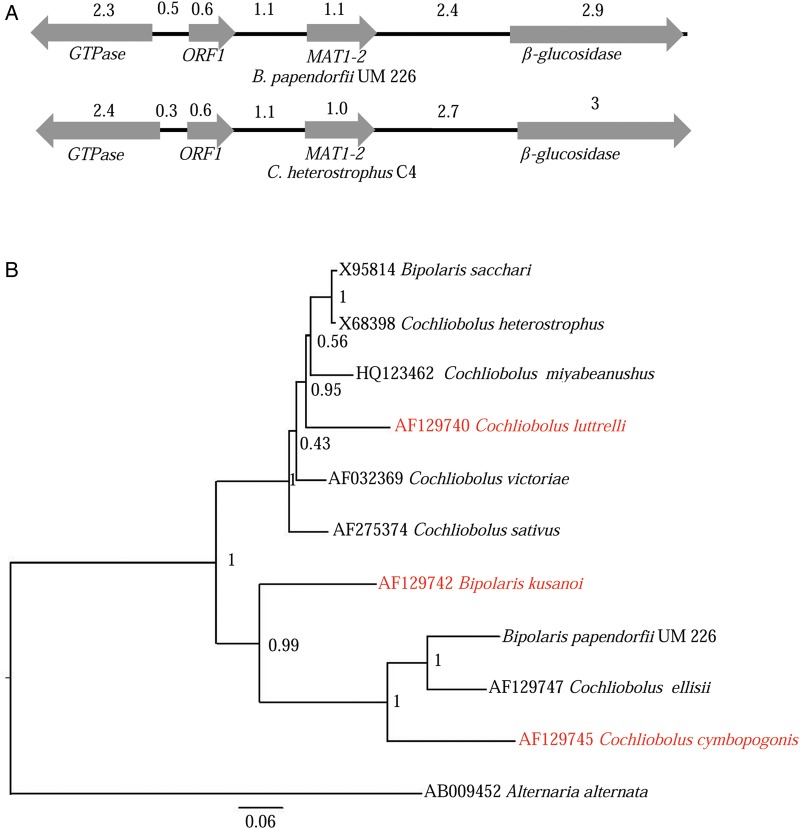
*MAT1-2* gene of *Bipolaris papendorfii* UM 226. (A) Schematic representation of major open reading frames (ORFs) identified in the *MAT* regions of *B. papendorfii* UM 226 and *Cochliobolus heterostrophus* C4. Numbers are in kilobases. (B) Bayesian phylogram generated based on *MAT1-2* nucleotide sequences. The tree is rooted with *Alternaria alternata* as outgroup. Numbers on the nodes indicate Bayesian posterior probability based on 100 sampling frequency for a total of 150,000 generations. This figure is available in black and white in print and in colour at *DNA Research* online.

Sexual reproduction is well characterized for most of the known *Cochliobolus* species.^29^ In *Cochliobolus* species, a single mating type locus (MAT) was important to regulate sexual development and production of sexual spores.^54^ The coexistence of heterothallic and homothallic species are common in the genus of *Cochliobolus*. Thus, sexual *Cochliobolus* species can be either heterothallic fungi that require two strains of opposite mating type to participate in the sexual process (*MAT1-1* or *MAT1-2* gene) or homothallic fungi that carry both *MAT1-1* and *MAT1-2* genes.^29^ In this study, we successfully identified a putative *MAT1-2* (UM226_825) gene but no *MAT1-1* gene in the UM 226 genome. UM226_825 shared a homology (81% identical) and domain architecture (DNA domain of the high mobility group, HMG domain) with the MAT-2 from *Curvularia ellisii*. The structural organization of 12 kb at the *B. papendorfii* UM 226 *MAT1-2* region was examined (Fig. 7a). As previously described,^6,54^ three other putative genes have been identified adjacent to the *MAT1-2*: (i) a β-glucosidase homolog gene (3′ flank of *MAT1-2*), (ii) a homolog of a yeast gene (ORF1) with unknown function (5′ flank of *MAT1-2*) and (iii) a GTPase-activating homolog gene (5′ flank of *MAT1-2*) (Fig. 7a). However, these genes were not involved in sexual reproduction.^54^ Turgeon *et al.*^54^ showed that the *MAT1-2* gene evolved at a faster rate than other nucleotide sequences in the genome of *Cochliobolus* species. In this study, a phylogenetic tree was built to reveal evolutionary relationships between *B. papendorfii* and other related species (Fig. 7b). *MAT1-2* genes from three homothallic fungi and six heterothallic fungi were included in the analysis (Supplementary Table S7). Based on the phylogenetic analysis, the *MAT1-2* genes can be divided into two groups, which agree well with the tree previously discussed (Fig. 2). *B. papendorfii MAT1-2* is tightly clustered together with the *MAT1-2* of heterothallic *C. ellisii*, which are separated from another group that consisted of virulent plant pathogens. The close relationship of asexual *B. papendorfii* to the sexual *C. ellisii* suggests that *B. papendorfii* can also be reproduced sexually in field.

### 3.8. Secondary metabolism

Fungal secondary metabolites are a double-edged sword. They are the source of medicinal biologically active compounds. They are also notorious mycotoxins that cause diseases on plants, animals and humans. Armed with the knowledge that secondary metabolite biosynthetic genes often occur in clusters with a key backbone gene.^55^ In this study, we used SMURF prediction to annotate potential gene cluster backbone enzymes in the *B. papendorfii* UM 226 genome. There are 32 gene clusters encoding core enzymes, including 19 genes encoding polyketide synthase (PKS) or PKS-like, 11 genes encoding non-ribosomal peptide synthetase (NRPS) or NRPS-like, a gene encoding polyketide synthase/non-ribosomal peptide synthetase hybrid (PKS-NRPS) and a gene encoding dimethylallyl tryptophan synthase (DMATS), that are important for the biosynthesis of melanin, bioactive compounds and mycotoxins. The only *DMATS* gene identified in *B. papendorfii* UM 226 is UM226_1500 (48% identical), which is often involved in the first committed step of ergot alkaloids biosynthesis.^56^ Ergot alkaloids are mycotoxins but also important natural pharmaceuticals for human use.^57^

Like most *Cochliobolus* plant pathogens reported,^6^ *B. papendorfii* UM 226 is enriched with multidomain NRPS encoding genes (NPS) and PKS genes. *NPS2* (UM226_2338, 74% identical), *NPS6* (UM226_3398, 87% identical), *NPS4* (UM226_5568, 82% identical) and *NPS10* (UM226_3258, 93% identical) orthologous genes found in *B. papendorfii* UM 226 were reported to be conserved across all *Cochliobolus* species.^29^ NPS2, NPS6, NPS4 and NPS10 harbour multiple domains, including AMP-binding domain, condensation domain, phosphopantetheine-binding domain and amino acid adenylation domain. NPS2 is likely responsible for the synthesis of siderophore and intracellular iron storage.^28^ All of the reported *Cochliobolus* pathogens are necrotrophs, which produce Host Selective Toxins (HSTs) to infect cereal crops.^58^ Like *C. carbonum* (*Bipolaris zeicola*), *B. papendorfii* has been reported to cause Corn Leaf Spot disease.^9^ Thus, it is not surprising that *B. papendorfii* UM 226 contains genes encoding HST. Interestingly, UM226_5568 shared a homology (69% identical) with the HC-toxin synthetase from *Pyrenophora tritici-repentis* (strain Pt-1C-BFP). As noted previously,^6^ the NPS4 encoded by UM226_5568 might be responsible for producing HC-toxin. In addition, four putative HC-toxin efflux carrier TOXAs were identified in the genome, including UM226_2212 (77% identical), UM226_5722 (74% identical), UM226_9947 (55% identical) and UM226_6817 (50% identical). These putative HC-toxin efflux carrier TOXAs have a potential role in self-protection against secreted HC-toxin.

Melanin production, a hallmark of dematiaceous fungi, is responsible for the protection of the fungi from UV irradiation, high temperatures as well as oxidants.^59^ *PKS18*, encoding a PKS for melanin biosynthesis, is conserved in all *Cochliobolus* plant pathogens.^29^ However, this *PKS18* gene was not identified in *B. papendorfii* UM 226, implying that that there must be other genes, such as PKS1 (UM226_1411, 93% identical), PKS2 (UM226_3388, 84% identical), PKS4 (UM226_8230, 79% identical), PKS7 (UM226_6241, 65% identical), PKS9 (UM226_7130, 77% identical) and PKS11 (UM226_7830, 85% identical) that can be responsible for melanin biosynthesis. Among these, *PKS1* (UM226_1411), *BRN1* (UM226_1408, 98%) and transcription factor *CMR1* (UM226_1409, 84%) are located in a close group, and their mutual orientation is the same as found in *C. heterostrophus* and *A. brassicicola*.^60^ Moreover, two important melanin biosynthetic genes, scytalone dehydratases (*SCD1*), UM226_59, (98% identical) and UM226_7826 (65% identical), were identified. PKS1, BRN1 and SCD1 are the key enzymes involved in the 1,8-dihydroxynaphthalene (DHN) biosynthesis pathway for the melanin biosynthesis.^60^ Transcription factor CMR1 plays an important role to control *BRN1* and *SCD1* genes expression during melanin production.^61^ Eliahu *et al.*^60^ indicated that the expression of *CMR1* is regulated by two mitogen-activated protein kinases (MAPKs) known as Mps1 and Chk1 for normal pigmentation; both enzymes were found in our genome. Two Mps1 (UM226_10400, 95% identical and UM226_10401, 93% identical) and two Chk1 (UM226_10509, 87% identical and UM226_10016, 50% identical) were identified. Overall, these findings suggested that like most of the dematiaceous fungi, melanin is biosynthesized via the DHN-melanin pathway, which is mediated by the transcription factor CMR1 and two signaling components, Mps1 and Chk1.

Terpenoids, also referred to as isoprenoids, are a diverse and the largest group of natural bioactive compounds. Many terpenoids such as the anticancer drug paclitaxel and the antimalarial drug Artemisinin are well recognized for human disease therapy.^62^ In this study, we identified 10 core enzymes involved in the mevalonate (MVA) pathway for terpenoid biosynthesis (Supplementary Table S8). The enzymes 3-hydroxy-3-methylglutaryl-coenzyme A reductase and acetyl-CoA C-acetyltransferase are encoded by two and four copies of the genes, respectively. The remaining eight enzymes are encoded by single-copy genes. Taken together, terpenoid backbone biosynthesis in *B. papendorfii* UM 226 might occur mainly through the MVA pathway.

Caffeine (1,3,7-trimethylxanthine), a purine alkaloid, which is one of the world's most popular psychoactive drug in pharmaceuticals, with an estimated consumption of 120,000 tons worldwide per year.^63^ Caffeine is a large environmental concern because of the production of millions tons of caffeine-containing waste products every year. There are few publication about microorganisms capable of caffeine degradation have been isolated from different environments.^64,65^ Sauer *et al.*^66^ indicated that caffeine was degraded by cytochrome P450 enzymes in yeast, postulating that the catabolic pathway might be more similar to that in animals. In this work, we searched the *B. papendorfii* UM226 genome for potential genes involved in caffeine metabolism. Two cytochrome P450 enzymes (UM226_867, 78% identical and UM226_7104, 39% identical) were identified in the genome based on the KEGG metabolic pathway analysis. In addition, we also found two cytochrome P450 2E1 (UM226_1044, 81% identical and UM226_4157, 79% identical) that catalyze caffeine metabolism via N-demethylation pathway in human liver cells. The production of methylxanthines during the course of caffeine degradation can be further converted to the methyluric acids by xanthine oxidase.^64^ In this work, two xanthine oxidases (UM226_7607, 92% identical and UM226_7880, 86% identical) were found in the *B. papendorfii* UM 226 genome. Moreover, we also identified an uricase (UM226_10622, 83% identical) that might responsible to convert free xanthine into simpler compounds.^64^ This suggests that *B. papendorfii* UM 226 may be a potential caffeine-resistant strain of fungus.

In this study, we successfully generated a high-quality draft genome sequence of *B. papendorfii* UM 226 using hybrid assembly of Illumina and PacBio sequencing technologies. This is the first genome assembly of *B. papendorfii*, which allows us to carry out phylogenetic and comparative genomic analyses. Phylogenomic analysis revealed that *B. papendorfii* UM 226 tightly clustered with *C. lunatus* m118, which is consistent with the multilocus phylogenetic analysis. However, at this stage of knowledge, the long standing confusion in the taxonomic grouping of *Bipolaris*, *Curvularia* and *Cochliobolus* remains unsolved. In this work, we identified a unique gene encoding LysM-containing protein. The LysM-containing protein has three LysM domains which are predicted to bind chitin to perturb chitin-induced immunity from the host. Furthermore, *B. papendorfii* UM226 are enriched with putative DMAT, PKS and NRPS encoding genes. The presence of these key enzymes will serve as a landmark for future discovery of bioactive molecules as well as mycotoxins. We hope that the dissection of the *B. papendorfii* UM226 genome sequence and in-depth analysis of the genome content will help to decipher its basic biology and provide a valuable platform for future fungal research.

## 4. Availability

This Whole Genome Shotgun project has been deposited at DDBJ/EMBL/GenBank under the accession JXCC00000000. The version described in this paper is version JXCC01000000.

## Supplementary data

Supplementary data are available at www.dnaresearch.oxfordjournals.org.

## Funding

This study was supported by High Impact Research MoE Grant UM.C/625/1/HIR/MOHE/MED/31 (No. H-20001-00-E000070). Funding to pay the Open Access publication charges for this article was provided by High Impact Research MoE Grant UM.C/625/1/HIR/MOHE/MED/31 (No. H-20001-00-E000070).

## Supplementary Material

Supplementary Data
