# Supplementary material for: Dissecting the fungal biology of Bipolaris papendorfii: from phylogenetic to comparative genomic analysis
Source: DNA Res. 2015 Apr 27;22(3):219–32. doi: 10.1093/dnares/dsv007 (PMC4463846; doi:10.1093/dnares/dsv007)
Supplement: Supplementary Data [file supp_22_3_219__index.html]

Dissecting the fungal biology of Bipolaris papendorfii: from phylogenetic to comparative genomic analysis — Supplementary Data 

# Dissecting the fungal biology of *Bipolaris papendorfii*: from phylogenetic to comparative genomic analysis

## Supplementary Data

Supplementary Data

**Files in this Data Supplement:**

- Supplementary Table 1 - xlsx file
- Supplementary Table 2 - xlsx file
- Supplementary Table 3 - xlsx file
- Supplementary Table 4 - xlsx file
- Supplementary Table 5 - xlsx file
- Supplementary Table 6 - xlsx file
- Supplementary Table 7 - xlsx file
- Supplementary Table 8 - xlsx file
- Supplementary Figure 1 - tif file
