# Supplementary figures and images for: Dissecting the fungal biology of Bipolaris papendorfii: from phylogenetic to comparative genomic analysis
Source: DNA Res. 2015 Apr 27;22(3):219–32. doi: 10.1093/dnares/dsv007 (PMC4463846; doi:10.1093/dnares/dsv007)

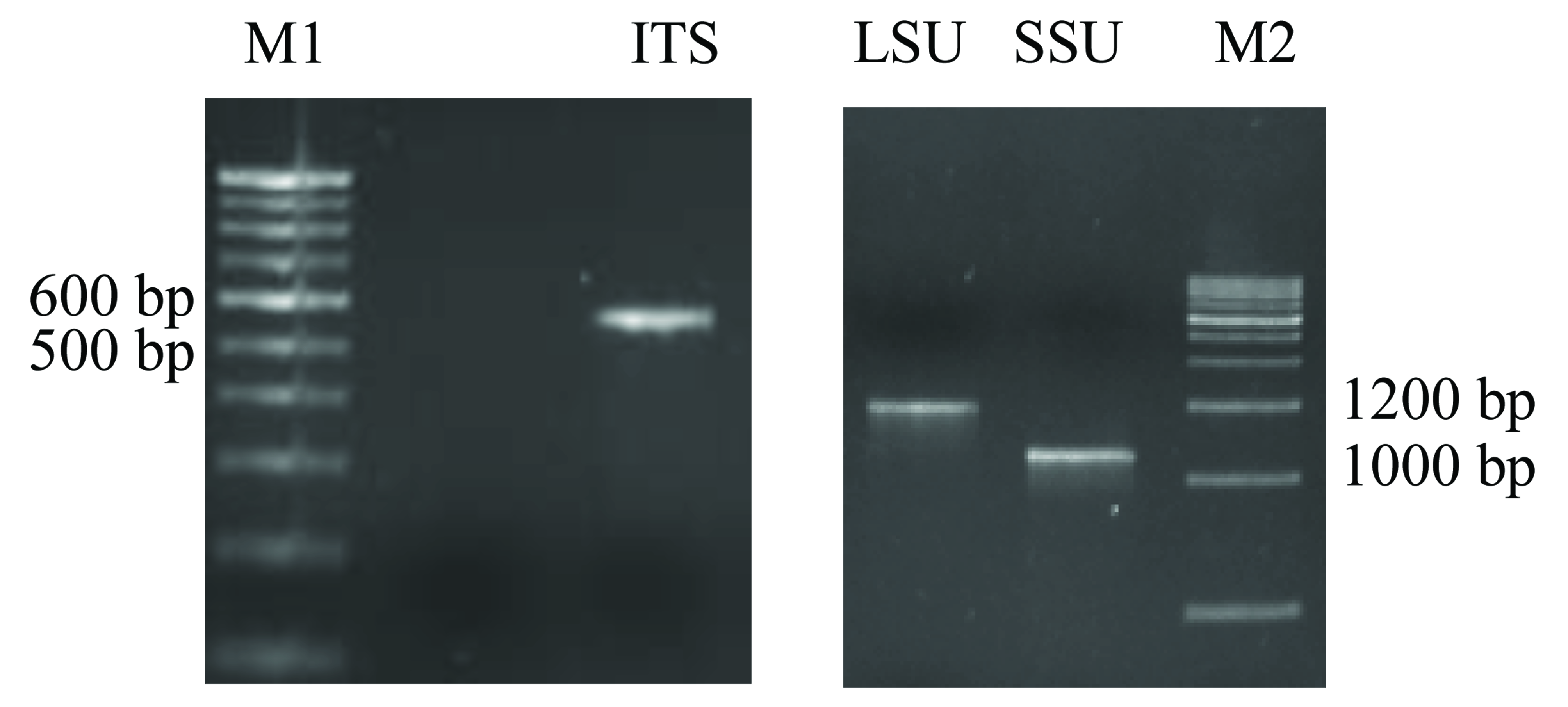

Supplement: Supplementary Data [file supp_dsv007_dsv007supp_fig1.tif]
